# PERSPECTIVES OF THE EM-SART: INTERVIEWS WITH ADULT PROTECTIVE SERVICES CLIENTS

**DOI:** 10.1093/geroni/igac059.1596

**Published:** 2022-12-20

**Authors:** Jason Burnett, Kristin Lees Haggerty, Randi Campetti, Rebecca Stoeckle

**Affiliations:** The University of Texas McGovern Medical School at Houston, Houston, Texas, United States; Education Development Center, Inc., Waltham, Massachusetts, United States; Education Development Center, Waltham, Massachusetts, United States; Education Development Center, Inc., Waltham, Massachusetts, United States

## Abstract

Older adults who experience mistreatment are more likely to visit emergency departments (ED), yet screening and response protocols in these health care settings are sorely lacking. Protocols designed with the mistreated older adult’s perspective in mind are needed to maximize efficacy and effectiveness. In this study, 18 mistreated older adults completed semi-structured perspective interviews regarding the ED Elder Mistreatment Screening and Response Tool (EM-SART). The findings highlight the importance of training healthcare staff to ask elder mistreatment (EM) questions in a preset context and to ask EM questions with empathy, concern, privacy, and clarity. Participants also stressed the desire to be reported to Adult Protective Services, but to also be included in the safety planning. These findings have direct implications for training health care workers to screen and respond to EM in the ED.

